# Predictors of postpartum family planning in Rwanda: the influence of male involvement and healthcare experience

**DOI:** 10.1186/s12905-021-01253-0

**Published:** 2021-03-19

**Authors:** Pamela Williams, Nicole Santos, Hana Azman-Firdaus, Sabine Musange, Dilys Walker, Felix Sayinzoga, Yea-Hung Chen

**Affiliations:** 1 Institute for Global Health Sciences, University of California San Francisco, San Francisco, CA USA; 2 Global Health Sciences, AIDS Research Institute and Cochrane HIV/AIDS Group, University of California San Francisco, San Francisco, CA USA; 3 School of Public Health, College of Medicine and Health Sciences, University of Rwanda, Kigali, Rwanda; 4 Department of Obstetrics, Gynecology, and Reproductive Sciences, University of California San Francisco, San Francisco, CA USA; 5 Maternal, Child and Community Health Division, Rwanda Ministry of Health, Rwanda Biomedical Center, Kigali, Rwanda; 6 Center for Public Health Research, San Francisco Department of Public Health, San Francisco, CA USA

**Keywords:** Postpartum family planning, Reproductive health, Birth spacing, Male involvement, Maternal health

## Abstract

**Background:**

Strengthened efforts in postpartum family planning (PPFP) is a key priority to accelerate progress in reproductive, maternal, newborn, and child health outcomes. This secondary data analysis explores factors associated with PPFP uptake in Rwanda. The purpose of this study was to explore variables that may influence PPFP use for postpartum women in Rwanda including health facility type, respectful maternity care, locus of control, and mental health status.

**Methods:**

This secondary analysis of data from a cluster randomized control trial used information abstracted from questionnaires administered to women (≥ 15 years of age) at two time points—one during pregnancy (baseline) and one after delivery of the baby (follow-up). The dependent variable, PPFP uptake, was evaluated against the independent variables: respectful care, locus of control, and mental health status. These data were abstracted from linked questionnaires completed from January 2017 to February 2019. The sample size provided 97% power to detect a change at a 95% significance level with a sample size of 640 at a 15% effect size. Chi-square testing was applied for the bivariate analyses. A logistic regression model using the generalized linear model function was performed; odds ratio and adjusted (by age group and education group) odds ratio with 95% confidence interval were reported.

**Results:**

Of the 646 respondents, although 92% reported not wanting another pregnancy within the next year, 72% used PPFP. Antenatal care wait time (*p* =  < 0.01; Adj OR (Adj 95% CI) 21–40 min: 2.35 (1.46,3.79); 41–60 min: 1.50 (0.84,2.69); 61–450 min: 5.42 (2.86,10.75) and reporting joint healthcare decision-making between the woman and her partner (male) (*p* = 0.04; Adj OR (Adj 95% CI) husband/partner: 0.59 (0.35,0.97); mother and partner jointly: 1.06 (0.66,1.72) were associated with PPFP uptake.

**Conclusions:**

These results illustrate that partner (male) involvement and improved quality of maternal health services may improve PPFP utilization in Rwanda.

**Supplementary Information:**

The online version contains supplementary material available at 10.1186/s12905-021-01253-0.

## Background

Worldwide, there is significant unmet need for family planning in the postpartum period. Postpartum family planning (PPFP) is defined as the prevention of unintended and closely spaced pregnancies for the first 12 months following childbirth [1]. The average desired family size varies, but regardless, women typically spend about thirty years (three-quarters of their reproductive lives) trying to prevent pregnancy [2]. Distinctly from nulliparous women, parous women prioritize the ability to manage inter-pregnancy intervals and halt childbearing when desired in addition to avoiding unintended pregnancies [3, 4]. Thus, distinctive interventions for this population, compared to women who have not been pregnant, are necessary. More than 90% of women globally report a desire to space or limit additional pregnancies postpartum, however, 61% do not use contraception [5].

PPFP is one of the most effective methods to improve reproductive, maternal, newborn, and child health (RMNCH) outcomes and prevent unintended or closely spaced pregnancies following childbirth [3, 6–8]. The World Health Organization (WHO) distinguishes the postpartum period as the most imperative, yet overlooked stage in the lives of mothers and babies and advises at least 24 months between a birth and the subsequent pregnancy [8, 9].

Countries with high rates of facility-based deliveries provide an opportunity to address the unmet need for PPFP by offering contraceptive counseling prior to discharge [8]. One such country where PPFP has potential for great impact is the Republic of Rwanda. While the nation has dramatically reduced its maternal and newborn mortality rates and increased the use of modern contraception [10–12], 26% of Rwandan women have unmet family planning needs in their first year postpartum [13] and near one-half of births are conceived before the recommended interval of 24 months [14]. Contraception uptake postpartum in Rwanda has potential to prevent one in three maternal deaths [11]. Thus, accelerating national progress in RMNCH includes strengthened efforts in the area of PPFP [12].

Numerous infrastructure and health workforce developments have provided a foundation to facilitate PPFP uptake in Rwanda including: (1) public education campaigns [11, 15]; (2) health workforce reinforcements of skilled birth attendants and community health workers (CHWs) [11, 12, 15–17]; and (3) strengthened population-healthcare links through the mHealth system and an updated postnatal care (PNC) framework (Additional file 1: Fig. S1 [18–22]). This PNC framework, distributed in 2016, provides infrastructure for PPFP counseling [23–27]. Currently, prior to discharge at PNC 1, nearly a quarter of women enroll in a family planning method and two-thirds plan to engage in PPFP at a subsequent visit [8].

Multiple factors play a role in PPFP decision-making which must be further understood to deconstruct the facilitators and barriers to PPFP use. Health facility type, respectful maternity care, locus of control, and mental health status are potential elements that influence PPFP uptake. First, faith-based health facilities compose 30% of Rwanda’s healthcare system and supply critical gaps in care [28, 29]. Some denominations of faith-based facilities offer natural methods only (rhythm beads); “more effective” family planning options remain absent at these facilities leading to possible gaps in PPFP [30, 31]. Second, various attributes of respectful service delivery are central to patients’ notions of quality [32–34] and influences PPFP uptake, but limited data exist on this correlation [35]. Third, the locus of control denotes the extent to which an individual perceives authority over events in their lives [36]. An individual with an *internal* locus of control is empowered, they perceive authority over their life experiences; an *external* locus of control results in fault of outside forces for life events, the individual perceives powerlessness [36]. Research in East Africa and Rwanda illustrates the association between locus of control and utilization of PNC [18, 37–39] and thus increased likelihood of PPFP utilization [36]. Lastly, poor maternal mental health has been associated with preterm and low birth weight [40], substandard breastfeeding and immunization coverage [41], being underweight or stunted [42], increased rates of diarrhea and febrile disease [41, 43], and negative effects on child development [41]. Mental health status, except in the circumstance of psychiatric episodes [44], and engagement in PPFP practices, have not yet been explored. This work provides insights into how these factors may play a role in PPFP decision-making and uptake.

## Methods

### Study design and participants

This sub-analysis was conducted within the Preterm Birth Initiative (PTBi) Rwanda study [45], a collaboration among University of California San Francisco (UCSF), University of Rwanda, the Rwanda Ministry of Health (MOH), and the Rwanda Biomedical Center. As part of a cluster randomized control trial that tested a group model of antenatal (ANC) and PNC service delivery (NCT03154177), questionnaires were administered in person by study-trained data collectors to a cohort of women at two time points—one during pregnancy (baseline) and one after delivery of the baby (follow-up). The parent study included 36 health centers across five districts in rural and urban settings. Inclusion in the primary analysis of the parent study stated that participants must: (1) Be a minimum age of 15 years at the time of enrollment, (2) Attend the first ANC visit before 24 completed weeks of pregnancy, (3) Attend more than one ANC visit at one of the 36 study facilities, and 4) Consent to participate in the study and follow-up. Additional methods specific to the parent study are reported in the parent study protocol publication [46]. The dependent variable, PPFP uptake, was evaluated against the independent variables: respectful care, locus of control, and mental health status.

### Sampling: parent study and sub-analysis

The baseline questionnaire was answered by a sub-set of parent study participants across trial arms made up of a convenience sample of the first five women to present for ANC per month. Similarly, the follow-up questionnaire was administered to a convenience sample of those who presented at the health center with newborns approximately six weeks after birth. Participants who completed both the baseline and follow-up questionnaire data, linked by a study key and were completed from January 2017 to February 2019, were included in this sub-analysis. The parent study obtained approval with both UCSF and University of Rwanda National Ethics Council institutional review boards. Participants completed written informed consent forms or selected to have it read aloud. This study protocol was reviewed and approved by the Rwanda National Ethics Committee (No 0034/RNEC/2017) and the UCSF Institutional Review Board (No 16-21177).

### Data abstraction and analysis

Variables were defined by collated questions collaboratively selected by PTBi. The study variables are: respectful service, locus of control, and mental health status. The survey questions selected for this secondary analysis, respective predictors and outcomes, considerations to determine appropriate scoring of questionnaires, and methods to determine outcomes are detailed in Additional file 2: Table S1. Cronbach’s alpha scores (scale reliability) for respectful care and mental health question groupings are reported in Additional file 2: Table S1. The additional Locus of Control and Edinburgh Postnatal Depression Scale questionnaires are validated and were scored according to their respective scales [47–49]. Self-reported PPFP type was categorized as either more effective (sterilization, intrauterine device (IUD), sub-dermal implants, injectables) or less effective (condoms used alone, emergency contraception, or natural family planning), as supported in the literature [30]. Education level, occupation, household income, food security, and middle upper arm circumference (MUAC) were used as socioeconomic status indicators [50, 51]. A five-point Likert Scale was used for survey questions with the exception of one question on the locus of control questionnaire and one question on the Edinburgh Postnatal Depression Scale which uses the 4-point standard. RStudio 1.0.153 statistical software was used. The sample size provides 97% power to detect a change at a 95% significance level with a sample size of 640 at a 15% effect size. We applied non-parametric and parametric testing for all bivariate analysis where appropriate. The association between PPFP and most independent variables were evaluated with a Chi-square test; variables tested were categorical with more than two levels. Odds ratio and adjusted (by age group and education group) odds ratio with 95% confidence interval were performed and reported. Controlling for confounding was determined by multiple logistic regression for both the age and education groups. A *p* value of < 0.05 was considered statistically significant.

## Results

A total of 646 survey respondents completed the baseline and follow-up questionnaires. Demographic and partner communication data were available for 94% of survey respondents (Table 1). Most respondents (50%) were 26–35 years of age and had not progressed beyond mid-secondary education (88%). Most respondents (66%) had three or fewer previous births. The majority of women reported they could discuss matters related to their pregnancy with their partner (86%).

**Table 1:** Demographic variables of interest and partner communication among survey participants in Rwanda at baseline (n = 606)^a^

	n	(%)
*Age (years)*		
17–25	198	(32.7)
26–35	302	(49.8)
36–46	105	(17.3)
*Education level*		
None or some primary	275	(45.2)
Completed primary or some secondary	262	(43.1)
Completed secondary or some/completed university	65	(10.7)
*Occupation*		
Farmer	439	(72.4)
Homemaker	66	(10.9)
Small business owner	40	(6.6)
Unemployed	36	(5.9)
Government or private employee	12	(2.0)
Student	5	(0.8)
Undeclared	8	(1.3)
Earn money for household	106	(18.06)
*Cooking fuel type*		
Wood/Other solid fuel	596	(98.5)
Kerosene/Gas	9	(1.5)
*Place for cooking*		
Indoor	110	(18.6)
Outdoor	481	(81.4)
*Food security: food availability in last month*^b^		
Stressed to find enough food for family	353	(58.1)
Missed food due to affordability	331	(54.4)
Stressed to run out of food	333	(54.8)
Run out of food before expected	289	(47.6)
Gone whole day with no food	140	(23.3)
*Middle upper arm circumference (MUAC)*^c^		
< 21 cm	7	(1.4)
> 21 cm	484	(94.3)
*Tobacco use/exposure*		
Smoking while pregnant	5	(0.8)
Smoking in home	37	(6.1)
Alcohol use during pregnancy	84	(13.8)
*HIV test result*		
Positive	8	(1.6)
Negative	480	(97.8)
Unknown	3	(0.6)
History of diabetes	4	(0.8)
History of hypertension	5	(1.0)
Gravidity^d^		
1	122	(23.8)
2	103	(20.1)
3	99	(19.3)
4	87	(17.0)
5 +	102	(19.9)
*Parity*^e^		
0	133	(25.9)
1	107	(20.9)
2	99	(19.3)
3	84	(16.4)
4	37	(7.2)
5 +	53	(10.3)
Attended PNC with previous pregnancy^f^	125	(24.2)
Can discuss any matter related to pregnancy openly with partner	521	(86.1)

^a^Information not available for some participants; ^b^Yes/No statement, yes answers listed; ^c^The cut-off of < 21 cm for the MUAC variable identifies pregnant women with nutrition intake most at risk for low birth weight deliveries; < 23 cm is considered a conservative estimate [52]; ^d^ includes those ended in abortion; individuals pregnant with twins recorded as gravid 1; ^e^ most often women do not include stillbirths and infants who died shortly after birth; ^f^calculated only for women with reported parity ≥ 1; PNC = postnatal care

### PPFP uptake and variable correlation

PPFP uptake prevalence and related variable correlations were explored using data from the follow-up survey (Table 2). The majority of respondents (92%) did not want a pregnancy within the next year (Table 3) and 72% utilized a family planning method within 12 weeks postpartum. Of those using PPFP, 66% were using a “more effective” method. Those not using family planning were asked why they had not selected a method; 53% of those asked this question provided an answer. Half of those responding “other” cited waiting until their child was older before starting PPFP.

**Table 2:** Postpartum family planning (PPFP) usage among survey participants in Rwanda at follow-up (n = 646)^a^

	n	(%)
*Currently using family planning postpartum*		
Yes	465	(72.0)
No	181	(28.0)
*Of those using family planning, type (n = 465)*		
Implant^b^	209	(32.5)
Injectable^b^	64	(10.1)
Pill/oral^b^	55	(8.7)
Breastfeeding/lactational amenorrhea	54	(8.5)
Condom	38	(5.9)
Other^c^	34	(5.7)
No response	185	(28.6)
*Of those not using family planning, reason (n = 181)*		
Did not know options, was not counseled	19	(10.5)
Could not decide	28	(15.5)
Other	50	(27.6)

^a^Questions posed to participants during ANC and/or within 12 weeks of birth; ^b^ Classified as “more effective” family planning method(30); ^c^ method usage prevalence < 5% grouped as “Other” consists of: Intrauterine device^b^, Standard days or rhythm, Withdrawal, Sterilization^b^, and Emergency contraception

**Table 3:** The association of uptake of postpartum family planning (PPFP) and healthcare experience variables among survey participants in Rwanda (Total n = 610)^a, b^

	Yes	(%)	No	(%)	*p* value	OR	95% CI	Adj OR	Adj 95% CI
Desire for pregnancy < 1 year									
Yes (n = 28)	21	(75.0)	7	(25.0)	< .01*	Ref	Ref	Ref	Ref
No (n = 560)	412	(73.6)	148	(26.4)		0.92	0.35,2.12	0.92	0.35,2.10
Undecided (n = 19)	4	(21.1)	15	(78.9)		0.08	0.02,0.33	0.09	0.02,0.32
No response (n = 3)	3	(100.0)	0	(0.0)					
Health facility designation					0.53				
Government (n = 468)	341	(72.86)	127	(27.14)		Ref	Ref	Ref	Ref
Faith-based (limited family planning services) (n = 142)	99	(69.72)	43	(30.28)		1.17	0.77,1.75	1.17	0.77,1.76
Respectful care					0.78				
Respectful care experienced by mother (n = 261)^a^	185	(70.88)	76	(29.11)		Ref	Ref	Ref	Ref
Respectful care not experienced (n = 346)	253	(73.12)	93	(26.88)		1.12	0.78,1.60	1.12	0.77,1.59
No response^2^ (n = 3)	2	(66.67)	1	(33.33)					
ANC wait time					< 0.01*				
0–20 min (n = 113)	63	(55.75)	50	(44.25)		Ref	Ref	Ref	Ref
21–40 min (n = 258)	191	(74.03)	67	(25.97)		2.26	1.42,3.60	2.35	1.46,3.79
41–60 min (n = 88)	58	(65.90)	30	(34.09)		1.53	0.87,2.74	1.50	0.84,2.69
61–450 min (n = 119)	104	(87.39)	15	(12.60)		5.50	2.91,10.91	5.42	2.86,10.75
No response (n = 32)	24	(75.00)	8	(25.00)					
Reported ANC attendance difficulty					0.29				
Yes (n = 143)	99	(69.23)	44	(30.77)		Ref	Ref	Ref	Ref
No (n = 457)	335	(73.20)	122	(26.70)		1.22	0.80,1.83	1.23	0.81,1.86
Unsure and No response (n = 10)	4	(40.00)	6	(60.00)		0.30	0.04,1.85	0.31	0.04,1.91
Locus of control					0.15				
Internal locus of control (n = 573)^c^	416	(72.60)	157	(27.40)		Ref	Ref	Ref	Ref
External locus of control (n = 28)	17	(60.71)	11	(39.29)		0.58	0.27,1.31	0.56	0.27,1.29
No response (n = 9)	7	(77.78)	2	(22.22)					
Makes healthcare decisions for mother and newborn					0.04*				
Mother (n = 124)	93	(75.00)	31	(25.00)		Ref	Ref	Ref	Ref
Husband/partner (n = 1187)	119	(63.63)	68	(36.36)		0.58	0.35,0.95	0.59	0.35,0.97
Mother and partner jointly (n = 297)	226	(76.09)	71	(23.91)		1.06	0.64,1.71	1.06	0.66,1.72
No response (n = 1)	1	(100.00)	0	(0.00)					
Perceived stress scale					0.08				
Moderate or low perceived stress (n = 194)	150	(77.31)	44	(22.68)		Ref	Ref	Ref	Ref
High perceived stress (n = 410)	279	(69.40)	131	(30.60)		0.67	0.44,0.98	0.67	0.45,0.99
No response (n = 15)	12	(80.00)	3	(20.00)					
Edinburgh postnatal depression scale					0.81				
“Normal” range of postpartum feelings (n = 28)	21	(75.00)	7	(25.00)		Ref	Ref	Ref	Ref
Out of normal range, advised to seek services (n = 552)	374	(71.65)	148	(28.35)		0.84	0.44,1.52	0.84	0.44,1.53
No response (n = 60)	45	(75.00)	15	(25.00)					

ANC = antenatal care; ^a^Questions posed to participants during ANC and/or within 12 weeks of birth; ^b^One or more values missing, subset of total sample; ^c^Participants’ responses showed cumulative score of positive internal locus of control; * indicates statistical significance < 0.05

### Association of uptake of PPFP and healthcare experience variables: respectful care, locus of control, and mental health

Women from the study sample attended 30 different primary care facilities, of which 77% were government public institutions and 17% were operated by faith-based organizations offering no “more effective” family planning methods (Table 3). The association between uptake of PPFP and health facility type was not statistically significant.

Relationships between variables related to healthcare experience and PPFP uptake were evaluated. Respectful maternity care, reported ANC attendance difficulty, locus of control, and mental health measures were not associated with PPFP uptake. ANC wait time and the individual/partner identified as the healthcare decision maker for the mother and newborn were statistically associated with PPFP uptake. Antenatal care wait time (*p* =  < 0.01) and reporting a partner (male) as the healthcare decision-maker (*p* = 0.04) were associated with PPFP uptake. The adjusted odds ratios for antenatal care wait time implies there is a difference between those taking PPFP and those not, except for those in the 41–60 min wait time group (1.50/0.84, 2.69). The adjusted odds ratios for healthcare decision making variable implies there is no difference between the two groups for those reporting the mother and partner as joint decision makers (1.06/0.66, 1.72).

## Discussion

Among the postnatal Rwandan women who participated in this study, most did not want a pregnancy within one year of delivery, yet 44% failed to utilize PPFP or a “more effective” PPFP method. The aim of this study was to determine if PPFP use was influenced by the independent variables tested. These analyses suggest that ANC wait time and the mother or partner identified as the healthcare decision maker for the mother and newborn were influential elements of PPFP uptake.

### Opportunities exist for increased rates of PPFP uptake

Overall, family planning use in Rwanda is accepted and the true reasons for non-use are difficult to elicit [53]. The low response rate observed for participants asked the reason for no family planning use could be due to social desirability; participants may feel hesitant to respond to this question because of favorable attitudes towards, or a feeling of pressure, to use contraceptives. This portion of the verbal survey did not provide prompted answers, another potential factor in the low response rate. Literature shows that the mode of questionnaire administration can impact data quality [54] and a “prefer not to answer” choice could have provided more insight into this absence of data.

A bolstered focus to increased sensitization to the health risks of pregnancy within two years of birth can decrease adverse RMNCH health outcomes and improve PPFP uptake [55]. The CHW network has greatly supplemented the health workforce shortages in Rwanda [17]. Family planning discussions currently take place, however, specific teaching to the health benefits of birth spacing could act as a facilitator for PPFP uptake and education of “more effective” methods. In addition, healthcare personnel can include PPFP in ANC and PNC education to supplement exposure to the health benefits of both PNC and PPFP. The continued work of family planning education, such as in the form of group care, in conjunction with an emphasis to improve RMNCH health outcomes with respect to the WHO two-year recommended birth window, can support this effort through the use of mobile technologies and bundled services [56, 57].

### Partner involvement: locus of control and the healthcare decision maker

The role of the partner in PPFP uptake was highlighted in this study. Women who reported joint healthcare decision making with their partner (husband) slightly increases the odds of PPFP uptake (Adj OR 1.06), while healthcare decision making by the partner independently decreases the odds of PPFP uptake (Adj OR 0.59).Thus, the inclusion and sensitization of men in the ANC and PNC process is warranted. The importance of male partners has been called out in previous research [58] Another study in Rwanda reported similar results: women in male-headed households are 20% more likely to attend PNC and have a skilled birth attendant present at birth or deliver at a health facility [39]. It can be inferred that male buy-in for PPFP may promote better RMNCH outcomes in Rwanda. Striking a balance between male involvement while supporting women’s autonomy will be an important consideration for partner involvement.

While the formal definition of locus of control was applied consistently throughout the study, the relationship between locus of control and PPFP uptake is likely more complex than the scale can reflect. The designation of internal locus of control was allocated if the participant reported that either the mother or the mother/partner jointly made healthcare decisions. An external locus of control was designated if the partner (husband) was reported as the decision-maker. This binary scoring mechanism, mirroring the standardized scoring, was created through a Western lens; the evidence in this analysis implies that the significance and meaning in the context of Rwanda is different. This could explain the absence of statistical significance for this collated measure. The results from this study suggest when the man is identified as the decision-maker, healthcare seeking behavior is supported more than if the mother independently or jointly made the decision with her partner. Thus, the over-simplification to a correlation of decision-maker (internal locus of control) and PPFP uptake excludes important considerations across various cultures and norms. Literature suggests that locus of control in the context of care engagement and health outcomes must incorporate additional considerations to more accurately identify correlations to behavior [59, 60]. Within Rwanda’s environment of a nation of both gender progressiveness and a continued prevalence of traditional values, healthcare decision-making within partnerships should be given special consideration [61].

Generally, a gap remains in data and knowledge on the subject of faith-based organizations’ contribution to RMNCH healthcare delivery, particularly in low- and middle-income (LMIC) countries [62, 63]. Evidence exists evaluating the availability of family planning services at faith-based organizations; however, in the context of Rwanda, it is known that facilities run by some denominations do not offer “more effective” family planning methods [64]. The MOH in Rwanda has attempted to address the gap in family planning services accessibility through the establishment of health posts in these areas, however, whether health posts improve access remains unknown. No comparable research has been done on the role of faith-based organization type and resultant family planning use.

Some study limitations exist. The study sample was limited to the evaluation of women in Rwanda who access care at a public facility. However, this population is believed to be representative of Rwandan women because 85–97% of women deliver at a public healthcare facility [13]. Second, the follow-up period was extended to a 12-weeks after delivery timeline in an attempt to include participants that did not complete the questionnaire within the original 8-week time window. This extension could skew results due to recall bias and may be more representative of those who have strong health seeking behaviors. Third, sampling bias could be present as the sample includes only those who were able to present at the health facility. Lastly, some respondents may have completed the survey prior to PPFP counseling; individuals may have engaged in PPFP after completion of the survey.

### Additional study insights

Despite lack of statistical significance compared against PPFP uptake, this analysis provided critical insights to the postpartum population. More than half (58%) of respondents indicated not receiving respectful care (questions detailed in A2 Table). Evidence of absence of respectful care is well documented and the results here posit additional support to the importance of health systems strengthening to reduce overworked healthcare workers [32–34]. Some ANC wait time adjusted odds ratios results suggested a difference between the two groups (those engaged in PPFP and those not). The odds ratio of five for the highest ANC wait time group necessitates further research. Additionally, 23% reported difficulty attending ANC [65, 66]. Both factors could have influence in postpartum follow-up and thus utilization of PPFP. However, this analysis does not illuminate a block of wait time as more influential towards PPFP uptake over others. This analysis also revealed high reports of perceived stress (66%) and postnatal depression (85%). The effects of perceived stress and postnatal depression have been well documented, including in relation to family planning use [41, 43, 44]. The timing of questionnaire administration as well as integration of identification of these patients has potential for accelerated impact on early childhood growth, among other benefits.

## Conclusion

Rwanda’s great strides in RMNCH makes it a unique context in which to evaluate RMNCH health outcomes. Numerous infrastructure and health workforce developments have provided a foundation to help facilitate PPFP uptake. Although the majority of women do not want an immediate subsequent pregnancy, 44% fail to utilize PPFP or a “more effective” PPFP method. These results illustrate that a gap exists in the utilization of PPFP services. These analyses illustrated that numerous factors can influence PPFP uptake. Preliminary recommendations to improve PPFP uptake include: 1) ensure access to more effective family planning methods at all facility types; 2) improve the quality of ANC and PNC services with reduced wait time; 3) include education in maternal care curriculums on the benefits of birth spacing; and 4) create a space for the partner, and/or male involvement in RMNCH and PPFP.

## Supplementary Information

**Additional file 1**: **Figure S1**. Rwanda’s newly initiated postnatal care (PNC) framework (distributed 2016). * General condition assessments of mother: physical examination, hygiene/hand washing counseling, breastfeeding support ** Breastfeeding assessments and counseling: volume of milk, positioning, attachment, mother’s nutritional intake, concerns such as nipple pain, engorgement, and mastitis, and supportive breastfeeding environment at health center; + General condition assessments of infant: physical examination, weight monitoring, immunization confirmation, social smiling, visual fixing, hearing screen; ++ Abnormality assessments of infants: jaundice, thrush, nappy rash, constipation, diarrhea, colic, fever. ^ Verified by health center staff if needed; ^^ Immunizations: BCG, OPV, DTP or DTP-HepB-Hib, Pneumococcal Conjugate, Rotavirus (21)

**Additional file 2**: **Table S1**. Selected survey questions, their respective predictors and outcomes, considerations to determine appropriate scoring of questionnaires, and methods to determine results

## Data Availability

Data for this analysis are housed with UCSF's data publication service and can be found here: https://doi.org/10.7272/Q6D21VR2.

## Abbreviations

PPFP: Postpartum family planning
RMNCH: Reproductive, maternal, newborn, and child health
WHO: World Health Organization
CHWs: Community health workers
PNC: Postnatal care
PTBi: Preterm Birth Initiative
UCSF: University of California San Francisco
MOH: Ministry of Health
ANC: Antenatal care
IUD: Intrauterine device
MUAC: Middle upper arm circumference
LMIC: Low- and middle-income country

## References

[1] World Health Organization. Programming strategies for Postpartum Family Planning [Internet]. 2013. Available from: https://apps.who.int/iris/bitstream/handle/10665/93680/9789241506496_eng.pdf

[2] Finer LB, Zolna MR (2016). Declines in Unintended Pregnancy in the United States, 2008–2011. N Engl J Med [Internet]..

[3] Ankomah A (2011). Myths, misinformation, and communication about family planning and contraceptive use in Nigeria. Open Access J Contracept [Internet]..

[4] Borda MR, Winfrey W, McKaig C (2010). Return to sexual activity and modern family planning use in the extended postpartum period: an analysis of findings from seventeen countries. Afr J Reprod Health..

[5] Family Planning 2020. Postpartum Family Planning [Internet]. 2018. Available from: http://www.familyplanning2020.org/microsite/ppfp

[6] Winikoff B, Sullivan M (2017). Assessing the role of family planning in reducing maternal mortality. Stud Fam Plann [Internet]..

[7] Byrne A, Morgan A, Soto EJ, Dettrick Z (2012). Context-specific, evidence-based planning for scale-up of family planning services to increase progress to MDG 5: health systems research. Reprod Health [Internet]..

[8] High Impact Practices in Family Planning (HIPs). Immediate postpartum family planning: A key component of childbirth care. [Internet]. Washington, DC; 2017. Available from: https://www.fphighimpactpractices.org/wp-content/uploads/2017/10/Immediate-PPFP.pdf

[9] Report of a WHO Technical Consultation on Birth Spacing Department of Making Pregnancy Safer (MPS) Report of a WHO Technical Consultation on Birth Spacing. World Heal Organ [Internet]. 2017; Available from: http://apps.who.int/iris/bitstream/10665/69855/1/WHO_RHR_07.1_eng.pdf

[10] Maternal mortality ratio (modeled estimate, per 100,000 live births) | Data [Internet]. Available from: https://data.worldbank.org/indicator/SH.STA.MMRT?locations=RW

[11] Worley H. Rwanda’s Success In Improving Maternal Health [Internet]. Population Reference Bureau. 2015. Available from: http://www.prb.org/Publications/Articles/2015/rwanda-maternal-health.aspx

[12] WHO. Success Factors for Women’s and Children’s Health Rwanda. 2014.

[13] National Institute of Statistics of Rwanda. Rwanda DHS Survey [Internet]. 2016. Available from: https://dhsprogram.com/pubs/pdf/FR316/FR316.pdf

[14] Family Planning Policy. 2012; Available from: http://www.moh.gov.rw/fileadmin/templates/Docs/Rwanda-Family-Planning-Policy.pdf

[15] Overseas Development Institute. Delivering maternal health: Why is Rwanda doing better than Malawi, Niger and Uganda? [Internet]. 2012. Available from: https://www.odi.org/sites/odi.org.uk/files/odi-assets/publications-opinion-files/7696.pdf

[16] Musange S. Rwanda Principal Investigator, personal communication. 2018.

[17] Condo J, Mugeni C, Naughton B, Hall K, Tuazon MA, Omwega A (2014). Rwanda’s evolving community health worker system: a qualitative assessment of client and provider perspectives. Hum Resour Health [Internet]..

[18] Rwabufigiri BN, Mukamurigo J, Thomson DR, Hedt-Gautier BL, Semasaka JPS (2016). Factors associated with postnatal care utilisation in Rwanda: a secondary analysis of 2010 Demographic and Health Survey data. BMC Pregnancy Childbirth [Internet]..

[19] Iconsphere. Noun Project - Icons for Everything: Hospital [Internet]. 2018. Available from: https://thenounproject.com/

[20] Republic of Rwanda Ministry of Health Maternal and Child Health Division. National Postnatal Care Guideline for Mother and Newborn. 2016

[21] CreativeStall. Noun Project - Icons for Everything: Home. Available from: https://thenounproject.com/

[22] Rwanda Biomedical Center Institute Of HIV/AIDS Disease Prevention & Control VACCINE Preventable Diseases Division. Comprehensive multi-year plan 2013–2017. 2012; Available from: http://www.nationalplanningcycles.org/sites/default/files/country_docs/Rwanda/attachment_6_revised_cmyp_08.pdf

[23] WHO. Assisting community health workers in Rwanda MOH ’ s RapidSMS and mUbuzima [Internet]. Innovation Catalysts. 2010. Available from: http://apps.who.int/iris/bitstream/10665/92814/1/WHO_RHR_13.15_eng.pdf%0Ahttp//http://apps.who.int/iris/bitstream/10665/92814/1/WHO_RHR_13.15_eng.pdf

[24] 2016 Rwanda: Rwanda RapidSMS Impact Evaluation | Evaluation database | UNICEF [Internet]. 2017. Available from: https://www.unicef.org/evaldatabase/index_95032.html

[25] Training Guide for Community health Workers on Home-based Provision of Planning Service in Rwanda. 2018. Available from: http://www.moh.gov.rw/fileadmin/templates/cdc/2010_September_Family_Planning_Services_at_the_Village_Level.pdf

[26] Procedures Manual for the Rwand Health Management Information System (HMIS). 2010.

[27] Project TIHSS. Building Systems for Better Health, Rwanda 2009–2014. 2009.

[28] A Firm Foundation the PEPFAR Consultation on the Role of Faith-based Organizations in Sustaining Community and Country Leadership in the Response to HIV/AIDS. 2018. Available from: https://www.pepfar.gov/documents/organization/195614.pdf

[29] Maurice J (2015). Faith-based organisations bolster health care in Rwanda. Lancet [Internet]..

[30] Cohen CR, Grossman D, Onono M, Blat C, Newmann SJ, Burger RL, et al. Integration of family planning services into HIV care clinics: results one year after a cluster randomized controlled trial in Kenya. PLoS ONE [Internet]. 2017. 10.1371/journal.pone.0172992&type=printable 10.1371/journal.pone.0172992 PMC5362197 28328966

[31] A Common Cause: Faith-Based Organizations and Promoting Access to Family Planning in the Developing World | Guttmacher Institute [Internet]. 2018. Available from: https://www.guttmacher.org/gpr/2013/12/common-cause-faith-based-organizations-and-promoting-access-family-planning-developing

[32] Tessema GA, Streak Gomersall J, Mahmood MA, Laurence CO. Factors determining quality of care in family planning services in Africa: a systematic review of mixed evidence. Mortimer K, editor. PLoS One [Internet]. 2016;11(11):e0165627. 10.1371/journal.pone.0165627 10.1371/journal.pone.0165627 PMC5094662 27812124

[33] Keesara SR, Juma PA, Harper CC (2015). Why do women choose private over public facilities for family planning services? A qualitative study of post-partum women in an informal urban settlement in Kenya. BMC Health Serv Res [Internet]..

[34] Goer H (2010). Cruelty in maternity wards: fifty years later. J Perinat Educ [Internet]..

[35] Bowser D, Hill MPHK. Exploring Evidence for Disrespect and Abuse in Facility-Based Childbirth Report of a Landscape Analysis [Internet]. 2010. Available from: https://cdn2.sph.harvard.edu/wp-content/uploads/sites/32/2014/05/Exploring-Evidence-RMC_Bowser_rep_2010.pdf

[36] Locus of Control | Encyclopedia of Psychology [Internet]. 2017. Available from: https://psychcentral.com/encyclopedia/locus-of-control/

[37] Tesfahun F, Worku W, Fekadu M, Kifle M. Knowledge, Perception and utilization of postnatal care of mothers in Gondar Zuria District, Ethiopia: A Cross-Sectional Study. Matern Child Heal [Internet]. 2014. Available from: https://link.springer.com/content/pdf/10.1007%2Fs10995-014-1474-3.pdf 10.1007/s10995-014-1474-3 PMC4220106 24770953

[38] Sipsma H, Callands TA, Bradley E, Harris B, Johnson B, Hansen NB (2013). Healthcare utilisation and empowerment among women in Liberia. J Epidemiol Community Heal [Internet]..

[39] Jayaraman A, Chandrasekhar S, Gebreselassie T. DHS WORKING PAPERS DHS WORKING PAPERS Factors Affecting Maternal Health Care Seeking Behavior in Rwanda. 2008. Available from: https://www.dhsprogram.com/pubs/pdf/WP59/WP59.pdf

[40] Grote NK, Bridge JA, Gavin AR, Melville JL, Iyengar S, Katon WJ (2010). A Meta-analysis of Depression During Pregnancy and the Risk of Preterm Birth, Low Birth Weight, and Intrauterine Growth Restriction. Arch Gen Psychiatry [Internet]..

[41] Parsons CE, Young KS, Rochat TJ, Kringelbach ML, Stein A (2012). Postnatal depression and its effects on child development: a review of evidence from low- and middle-income countries. Br Med Bull [Internet]..

[42] Surkan PJ, Kennedy CE, Hurley KM, Black MM (2011). Maternal depression and early childhood growth in developing countries: systematic review and meta-analysis. Bull World Health Organ [Internet]..

[43] Guo N, Bindt C, Te Bonle M, Appiah-Poku J, Tomori C, Hinz R (2014). Mental health related determinants of parenting stress among urban mothers of young children—results from a birth-cohort study in Ghana and Côte d’Ivoire. BMC Psychiatry [Internet]..

[44] Peindl KS, Zolnik EJ, Wisner KL, Hanusa BH (1995). Effects of postpartum psychiatric illnesses on family planning. Int J Psychiatry Med [Internet]..

[45] Preterm Birth Initiative [Internet]. 2017. Available from: http://pretermbirth.ucsf.edu/

[46] Musange SF, Butrick E, Lundeen T, Santos N, Azman Firdaus H, Benitez A (2019). Group antenatal care versus standard antenatal care and effect on mean gestational age at birth in Rwanda: protocol for a cluster randomized controlled trial. Gates Open Res.

[47] Rotter J. The Locus of Control [Internet]. 1966 [cited 2018 Feb 4]. Available from: http://www.psych.uncc.edu/pagoolka/LocusofControl-intro.html

[48] Cox JL (1987). JM Holden Edinburgh postnatal depression scale (EPDS). Br J Psychiatry..

[49] O’hara MW (1987). Edinburgh Postnatal Depression Scale (EPDS) Scoring; Other Information. Br J Psychiatry [Internet]..

[50] De Onis M, Yip R, Mei Z (1997). The development of MUAC-for-age reference data recommended by a WHO Expert Committee. Bull World Health Organ.

[51] Ballard Kepple, A. W., & Cafiero, C. (2013). Technical Paper. Rome, FAO. TJ. The food insecurity experience scale: Developing a global standard for monitoring hunger worldwide. Technical Paper. 2013. FAO Rome.

[52] Ververs M-T, Antierens A, Sackl A, Staderini N, Captier V. Which anthropometric indicators identify a pregnant woman as acutely malnourished and predict adverse birth outcomes in the humanitarian context? PLoS Curr [Internet]. 2013;5. Available from: http://www.ncbi.nlm.nih.gov/pubmed/23787989 10.1371/currents.dis.54a8b618c1bc031ea140e3f2934599c8 PMC3682760 23787989

[53] Frederic TM, Phoibe K, Ntaganira J. Assessment of Knowledge, Attitudes, and Practice on Contraceptive Use among Women Attending Family Planning Services in Some Health Centers of Muhima District Hospital, Rwanda. Open Sci J [Internet]. 2017;2(3). Available from: https://osjournal.org/ojs/index.php/OSJ/article/view/978

[54] Bowling A (2005). Mode of questionnaire administration can have serious effects on data quality. J Public Health (Bangkok) [Internet].

[55] WHO. Postnatal care of the mother and newborn [Internet]. 2013. Available from: http://apps.who.int/iris/bitstream/10665/97603/1/9789241506649_eng.pdf?ua=1 24624481

[56] Hall CS, Fottrell E, Wilkinson S, Byass P (2014). Assessing the impact of mHealth interventions in low- and middle-income countries—what has been shown to work?. Glob Health Action [Internet]..

[57] INTEGRATED HEALTH SERVICES-WHAT AND WHY? Making health systems work [Internet]. [cited 2018 Sep 20]. Available from: http://www.who.int/healthsystems/technical_brief_final.pdf

[58] Brunie A, Tolley EE, Ngabo F, Wesson J, Chen M. Getting to 70%: Barriers to modern contraceptive use for women in Rwanda. Int J Gynecol Obstet [Internet]. 2013;123(SUPPL.1):e11–5. 10.1016/j.ijgo.2013.07.005 10.1016/j.ijgo.2013.07.005 23992658

[59] Wallston KA (2018). Hocus-Pocus, the Focus Isn’t Strictly on Locus: Rotter’s Social Learning Theory Modified for Health. Ther Reseanzh [Internet]..

[60] Bastani F, Hashemi S, Bastani N, Haghani H (2010). Impact of preconception health education on health locus of control and self-efficacy in women. East Mediterr Health J [Internet]..

[61] Abbott P. The Promise and the Reality: Women’s Rights in Rwanda [Internet]. 2015. Available from: http://ohrh.law.ox.ac.uk/wordpress/wp-content/uploads/2014/04/OxHRH-Working-Paper-Series-No-5-Abott-and-Malunda.pdf

[62] Kagawa RC, Anglemyer A, Montagu D. The Scale of Faith Based Organization Participation in Health Service Delivery in Developing Countries: Systemic Review and Meta-Analysis. Beck EJ, editor. PLoS One [Internet]. 2012;7(11):e48457 10.1371/journal.pone.0048457 PMC3495941 23152775

[63] Olivier J, Tsimpo C, Gemignani R, Shojo M, Coulombe H, Dimmock F (2015). Understanding the roles of faith-based health-care providers in Africa: review of the evidence with a focus on magnitude, reach, cost, and satisfaction. Lancet [Internet]..

[64] Barden-O’Fallon J (2017). Availability of family planning services and quality of counseling by faith-based organizations: a three country comparative analysis. Reprod Health [Internet]..

[65] Påfs J, Musafili A, Binder-Finnema P, Klingberg-Allvin M, Rulisa S, Essén B (2015). “They would never receive you without a husband”: Paradoxical barriers to antenatal care scale-up in Rwanda. Midwifery.

[66] Okedo-Alex IN, Akamike IC, Ezeanosike OB, Uneke CJ (2019). Determinants of antenatal care utilisation in sub-Saharan Africa: a systematic review. BMJ Open..

