# Supplementary material for: Predictors of postpartum family planning in Rwanda: the influence of male involvement and healthcare experience
Source: BMC Womens Health. 2021 Mar 19;21:112. doi: 10.1186/s12905-021-01253-0 (PMC7980651; doi:10.1186/s12905-021-01253-0)
Supplement: Supplementary file 1 — Additional file 1: Figure S1. Rwanda’s newly initiated postnatal care (PNC) framework (distributed 2016). * General condition assessments of mother: physical examination, hygiene/hand washing counseling, breastfeeding support ** Breastfeeding assessments and counseling: volume of milk, positioning, attachment, mother’s nutritional intake, concerns such as nipple pain, engorgement, and mastitis, and supportive breastfeeding environment at health center; + General condition assessments of infant: physical examination, weight monitoring, immunization confirmation, social smiling, visual fixing, hearing screen; ++ Abnormality assessments of infants: jaundice, thrush, nappy rash, constipation, diarrhea, colic, fever. ^ Verified by health center staff if needed; ^^ Immunizations: BCG, OPV, DTP or DTP-HepB-Hib, Pneumococcal Conjugate, Rotavirus (21) [file 12905_2021_1253_MOESM1_ESM.docx]

| **A1 Fig. Rwanda’s newly initiated postnatal care (PNC) framework** (distributed 2016) (17-20) | | | | |
| --- | --- | --- | --- | --- |
| Visit # and location | 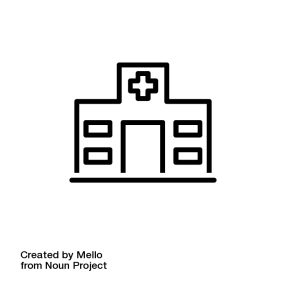PNC 1 | 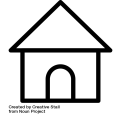PNC 2 | 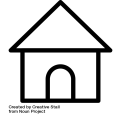PNC 3 | 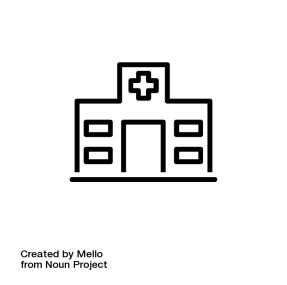PNC 4 |
| Conducted by | medical staff | community health worker | community health worker | medical staff |
| Timing | immediately prior to discharge following delivery | 48-72 hours after birth | Between days 7-14 after birth | Six weeks after birth |
| **Mother Content of Care Summary** | - general condition*, abnormality, emotional attachment, and breastfeeding** assessments  **-** physical examination  - screening for other conditions: cervical/ breast cancers, STIs  - family planning sensitization | - PNC 1 assessments  - blood pressure^^^  - gynecological examination^^^^  - leg thrombophlebitis  - Iron folate if needed  - nutrition counseling  - postpartum depression screening | - PNC 1 assessments  - check uterus, distended bladder  - review health history of family, woman | - PNC 1 assessments  - screening and medical history  - family planning sensitization |
| **Infant Content of Care Summary** | - general condition^+^, abnormality^++^, emotional attachment, and breastfeeding assessments  - identify pre-term, low birth weight  - Vitamin K  - immunizations  - promote skin to skin | - PNC 1 assessments | - PNC 1 assessments  - cord care  - review health history of baby | - PNC 1 assessments  - social smiling, visual fixing  - immunizations^ |
| * General condition assessments of mother: physical examination, hygiene/hand washing counseling, breastfeeding support  ** Breastfeeding assessments and counseling: volume of milk, positioning, attachment, mother’s nutritional intake, concerns such as nipple pain, engorgement, and mastitis, and supportive breastfeeding environment at health center;  ^+^ General condition assessments of infant: physical examination, weight monitoring, immunization confirmation, social smiling, visual fixing, hearing screen;  ^++^ Abnormality assessments of infants: jaundice, thrush, nappy rash, constipation, diarrhea, colic, fever  ^ Verified by health center staff if needed;  ^^ Immunizations: BCG, OPV, DTP or DTP-HepB-Hib, Pneumococcal Conjugate, Rotavirus (21) | | | | |
