# Supplementary material for: Predictors of postpartum family planning in Rwanda: the influence of male involvement and healthcare experience
Source: BMC Womens Health. 2021 Mar 19;21:112. doi: 10.1186/s12905-021-01253-0 (PMC7980651; doi:10.1186/s12905-021-01253-0)
Supplement: Supplementary file 2 — Additional file 2: Table S1. Selected survey questions, their respective predictors and outcomes, considerations to determine appropriate scoring of questionnaires, and methods to determine results [file 12905_2021_1253_MOESM2_ESM.docx]

| **A2 Table. Selected survey questions, their respective predictors and outcomes, considerations to determine appropriate scoring of questionnaires, and methods to determine results** | |
| --- | --- |
| **DEMOGRAPHIC VARIABLES** | |
| Predictors | Outcomes |
| Age  Education level  Occupation  Earn money for household  Cooking fuel type, environment  Food availability in last month  Tobacco use  Alcohol use  Can discuss any mater related to pregnancy openly with partner  Attended PNC during pregnancy  Gravidity  Parity  Middle upper arm circumference (MUAC)  HIV test result  History of diabetes  History of hypertension  Hemoglobin | Clustered demographic factors |
| **PPFP UPTAKE** | |
| 12-week Group  Health Related Behaviors (4 questions) 36-38 | Desire for pregnancy within 1 year ⇢ PPFP Y/N  PPFP Y ⇢ PPFP type  PPFP N ⇢ reason why |
| Health Related Behaviors (4 questions)  **23. Do you want to get pregnant again within the next year?**  1, Yes \| 2, No \| 3, Undecided  **24. Are you currently using a family planning method?**  1, Yes \| 2, No  **25. Why didn't you select a method of family planning?** (Allow the woman to respond to the question and then choose the best option from the answers)  1, I didn't know what the options were/I wasn't counseled/my provider didn't talk to me about it \| 2, I couldn't decide \| 3, I requested my preferred family planning method, but the provider didn't give it to me \| 4, Other (please specify below)  **26. What family planning method are you currently using?** (Allow the woman to respond to the question and then choose the best answer from the options)  1, Condoms \| 2, Pill/Oral contraceptives \| 3, Injectable \| 4, Implants \| 5, Intrauterine Device \| 6, Emergency contraception \| 7, Sterilization \| 8, Breastfeeding-Lactational Amenorrhea Method \| 9, Standard days or rhythm method \| 10, Withdrawal | |
| **HEALTH FACILITY TYPE** | |
| Health facility designation[65]  Government  Faith-based (limited family planning services) | Health facility designation ⇢ influence PPFP Y/N |
| **RESPECTFUL CARE, LOCUS OF CONTROL, MENTAL HEALTH STATUS** | |
| **Respectful Care**  Baseline  Satisfaction with care[66] (5 questions) 3,7,8,9,10  RS Group 1 (4 questions)  Cronbach's alpha = 0.82  RS Group 2 (5 questions)  Cronbach's alpha= 0.71  12-week  Satisfaction with care[66] (5 questions) 10,14,15,16,17  RS Group 3 (4 questions)  Cronbach's alpha = 0.82  RS Group 4 (5 questions)  Cronbach's alpha= 0.77 | Respectful care Y/N ⇢ PPFP Y/N |
| Satisfaction with care (5 questions)  Thinking about the antenatal care you received during this pregnancy and as best as you can remember, do you Strongly agree, Agree, Neither agree nor disagree, Disagree, or Strongly disagree with each of the following statements:  **10. I was greeted warmly.**  **14. The provider did not show me respect or act in respectful ways.**  **15. During my next pregnancy I would be happy to receive care similar to what I received during my antenatal care visit.**  **16. I would recommend to a friend to go to the same place I go for antenatal care.**  **17. I felt respected.** | |
| Respectful Care Scoring (5 questions, 5-point scale)  <8 = respectful care; if participant answered “strongly agree” or “agree,” scored as respectful care; one question (#7 baseline, #14 on follow-up) was reverse scored to account for double negative phrasing; both reverse score and direct interpretation are included | |
| Explanatory variable |  |
| ANC experience[66] (1 question) 9 | ANC wait time < 15 min ⇢ Respectful care Y ⇢ PPFP Y/N  ANC wait time > 16 min ⇢ Respectful care N ⇢ PPFP Y/N |
| ANC experience  **9. How many minutes (on average) did you have to wait for your ANC visit to begin once you had arrived at the health facility?** | |
| ANC experience (2 questions) 8,8a | Difficulty attending ANC Y/N ⇢ PPFP Y/N |
| ANC experience (2 questions)  **8. Did you experience any difficulties that prevented you from attending ANC at the health facility?**  1, Yes \| 2, No \| 3, Unsure  **8a. Why did you miss ANC visits, or why was it difficult to attend ANC visits?**  1, I didn't have enough time to attend ANC visits \| 2, I couldn't remember the day and/or time I was asked to return to the health facility \| 3, On the day and time I was asked to return for an ANC visit, I had to do something else and I couldn't go to the health facility \| 4, I didn't have transportation to the health facility \| 5, There was no one to care for my children while I attended ANC visits \| 6, I had too much work to do \| 7, My husband prevented me from attending ANC \| 8, My family members prevented me from attending ANC \| 9, I didn't like the ANC services offered at the health facility \| 10, I was treated poorly by health facility staff \| 11, I received ANC from a private facility instead \| 12, Other (please specify) | |
| **Locus of Control**  Baseline  LOC[67] (14 questions) 14-27  Follow-up  LOC (12 questions) 27-38 | 0 to 13 score range  ⇢ low score = internal control  ⇢ high score = external control[68]  LOC internal/external ⇢ PPFP Y/N |
| Locus of Control (14 questions)  Thinking about life generally, do you Strongly agree, Agree, Neither agree nor disagree, Disagree, or Strongly disagree with the following statements:  **14. I can talk to others about difficult subjects**  **15. I can let others know what I really think, even if it is different from them**  **16. I like to make decisions for myself**  **17. I am able to make decisions for myself**  Thinking about this pregnancy, do you Strongly agree, Agree, Neither agree nor disagree, Disagree, or Strongly disagree with the following statements:  **18. If I don’t understand something a provider is telling me I will tell them and ask them to explain a different way**  **19. There are things I can do to help prevent problems and keep myself and my baby healthy**  **20. I will talk with my husband/family about how to keep myself and our baby healthy.**  **21. I know how to recognize a problem with my pregnancy**  **22. I know what actions I will take if I think there is a problem with my pregnancy**  **23. I know how to recognize a problem with my newborn**  **24. I know what actions I will take if I think there is a problem with my newborn**  **25. I am good at making decisions related to the health of myself and my family**  **26. I feel confident I can ask my husband/partner to use a condom if I want him to use a condom.**  **27. Who will usually make decisions about health care for yourself and your newborn?**  1, You \| 2, Your husband/partner \| 3, You and your husband/partner jointly \| 4, Someone else (please specify below) | |
| Locus of Control Scoring (13 questions baseline, 11 questions follow-up, 5-point scale)  <35 = internal locus of control for baseline survey, <30 = internal locus of control for follow-up survey; if participant answered “strongly agree,” “agree,” or some “neutral” (average of 2.5 or less per question as “strongly agree” = 1, “agree” = 2, etc.) scored as internal to accommodate vague language for scoring of “internal” vs. “external”  #27 baseline, #38 follow-up LOC responses are recorded independently due to 4-point scale: “internal” LOC designated for answer values 1,3; “external” LOC designated for answer values 2,4 | |
| **Mental Health**  Baseline  Conduct sensitivity analysis comparing Group 1 PSS questions to Group 2 ⇢  PSS Group 1 (4 questions) 41,42,47,49  Cronbach's alpha = 0.65  PSS Group 2[67] (10 questions) 41-50  Cronbach's alpha = 0.84  Follow-up  Conduct sensitivity analysis comparing Group 3 PSS questions to Group 4 ⇢  PSS Group 3 (4 questions) 54,55,60,62  Cronbach's alpha = 0.79  PSS Group 4 (10 questions) 54-63  Cronbach's alpha = 0.89 | 0 to 40 score range  ⇢ 0-13 = low stress  ⇢ 14-26 = moderate stress  ⇢ 27-40 = high stress[69]  Low stress ⇢ PPFP Y/N  Moderate stress ⇢ PPFP Y/N  High stress ⇢ PPFP Y/N |
| Mental Health  Perceived Stress Scale (10 questions)  These questions ask you about your feelings and thoughts during the last month. The answer choices are Never, Rarely, Sometimes, Very Often, and Always.  **54. In the last month, how often have you been upset because of something that happened unexpectedly?**  **55. In the last month, how often have you felt that you were unable to control the important things in your life?**  **56. In the last month, how often have you felt nervous and “stressed”?**  **57. In the last month, how often have you felt confident about your ability to handle your personal problems?**  **58. In the last month, how often have you found that you could not cope with all the things that you had to do?**  **59. In the last month, how often have you felt that things were going your way?**  **60. In the last month, how often have you been able to control irritations in your life?**  **61. In the last month, how often have you felt that you were on top of things?**  **62. In the last month, how often have you been angered because of things that were outside of your control?**  **63. In the last month, how often have you felt difficulties were piling up so high that you could not overcome them?** | |
| Perceived Stress Scale Scoring (10 questions, 5-point scale)  <20 = lower stress; 4 questions reverse scored to accommodate wording (44,46,47,48 baseline and 57,59,60,61 follow-up); “never” and “rarely” scored as lower perceived stress | |
| Baseline  EPDS[70] (10 questions) 51-60  Follow-up  EPDS (10 questions) 64-73 | 0 to 3 score range each response  Reverse score 3, 5 to 10 (i.e., 3, 2, 1, and 0)  Scores added for total  Score > 12 or 13 = likely depressed[70]  Depressed Y/N ⇢ PPFP Y/N |
| Edinburgh Postnatal Depression Scale (10 questions)  As you have recently had a baby, we would like to know how you are feeling. For the next questions, please indicate the answer that comes closest to how you have felt in the past 7 days, now just how you feel today.  **64. I have been able to laugh and see the funny side of things.**  1, As much as I always could (0) \| 2, Not quite so much now (1 point) \| 3, Definitely not so much now (2 points) \| 4, Not at all (3 points)  **65. I have looked forward with enjoyment to things.**  1, As much as I ever did (0) \| 2, Rather less than I used to (1 point) \| 3, Definitely less than I used to (2 points) \| 4, Hardly at all (3 points)  **66. I have blamed myself unnecessarily when things went wrong.**  1, Yes, most of the time (3 points) \| 2, Yes, some of the time (2 points) \| 3, Not very often (1 point) \| 4, No, never (0)  **67. I have been anxious or worried for no good reason.**  1, No, not at all (0) \| 2, Hardly ever (1 point) \| 3, Yes, sometimes (2 points) \| 4, Yes, very often (3 points)  **68. I have felt scared or panicky for no good reason.**  1, Yes, quite a lot (3 points) \| 2, Yes, sometime (2 points) \| 3, No, not much (1 point) \| 4, No, not at all (0)  **69. Things have been getting on top of me.**  1, Yes, most of the time I haven't been able to cope at all (3 points) \| 2, Yes, sometimes I haven't been coping as well as usual (2 points) \| 3, No, most of the time I have coped quite well (1 point) \| 4, No, I have been coping as well as ever (0)  **70. I have been so unhappy that I have had difficulty sleeping.**  1, Yes, most of the time (3 points) \| 2, Yes, sometimes (2 points) \| 3, Not very often (1 point) \| 4, No, not at all (0)  **71. I have felt sad or miserable.**  1, Yes, most of the time (3 points) \| 2, Yes, quite often (2 points) \| 3, Not very often (1 point) \| 4, No, not at all (0)  **72. I have been so unhappy that I have been crying.**  1, Yes, most of the time (3 points) \| 2, Yes, quite often (2 points) \| 3, Not very often (1 point) \| 4, No, not at all (0)  **73. The thought of harming myself as occurred to me.**  1, Yes, most of the time (3 points) \| 2, Yes, quite often (2 points) \| 3, Only occasionally (1 point) \| 4, No, never (0) | |
| Edinburgh Postnatal Depression Scale Scoring (10 questions, 4-point scale)  <20 = normal emotions; selection of item 1 or 2; three questions reverse scored (items 53,55,60 baseline and 66,68,73 on follow-up) | |
